# Prevalence of Latent Tuberculosis Infection Among Nurses Working in Critical Areas at a Tertiary Care Hospital in Riyadh, Saudi Arabia

**DOI:** 10.7759/cureus.53389

**Published:** 2024-02-01

**Authors:** Nawaf M Aldhawyan, Abdulrahman K Alkhalifah, Mostafa Kofi, Yasser M Yousef, Abdulaziz A Alqahtani

**Affiliations:** 1 Family and Community Medicine, Prince Sultan Military Medical City, Riyadh, SAU

**Keywords:** healthcare workers, nursing staff, infectious diseases, tertiary care hospital, tuberculin skin test, prevalence, latent tuberculosis infection

## Abstract

Introduction

Tuberculosis is a critical health issue worldwide. Most infected persons are asymptomatic and categorized as having a latent tuberculosis infection (LTBI). Healthcare workers (HCWs) are more prone to being infected with tuberculosis and should be enrolled in a screening program for early detection.

Objectives

The study aims to estimate the prevalence of LTBI among nurses working in critical areas which include adult intensive care units, pediatric intensive care units, emergency departments, oncology departments, dialysis departments, tuberculosis labs, isolation rooms, and cardiac center intensive care units.

Methods

A record-based cross-sectional survey measured the prevalence of LTBI among nurses working in critical areas at Prince Sultan Military Medical City (PSMMC), Riyadh, Saudi Arabia. We reviewed the occupational health records of all nurses working in critical areas from June 1, 2021, to June 1, 2022. We recorded the data reviewed throughout the year in the Occupational Health Department at PSMMC. We excluded all participants with previously documented positive tuberculin skin test (TST) from the study. We analyzed the sociodemographic data, working years, working location, job title, and TST results.

Results

We included a total of 771 out of 2025 nurses in this study. Participants were mostly women (88%) and in the 26-35-year age group (67.7%). Most of the participants were originally from the Philippines (66.3%). The overall LTBI prevalence among nurses was 34.5%. The highest prevalence of LTBI was among nurses working in the cardiac intensive care unit (53.5%), and the lowest prevalence was among nurses working in the isolation department (8.9%; p-value <0.0001). Those who worked more in the hospital were significantly more infected with LTBI (p-value <0.04).

Conclusion

LTBI remains a significant health risk worldwide and in the Middle East as well as among HCWs. This underscores the necessity of comprehensive pre-hiring screening, annual screening, infection control protocols, and active management of HCWs with LTBI.

## Introduction

Tuberculosis is a critical health issue worldwide. It is predicted that more than 25% of the world population is infected with tuberculosis, the majority of which show no symptoms and are categorized as having a latent tuberculosis infection (LTBI) which is a major cause of death worldwide and ranked as the second main cause of death from infectious causes. Tuberculosis is transmitted via the inhalation of infectious aerosolized droplets. Healthcare workers (HCWs) are more prone to be infected with LTBI than the general population and should be enrolled in a tuberculosis screening program for the early detection of LTBI [1-3].

Although only 10% of infected people with *Mycobacterium tuberculosis* develop active disease, particularly in the early few years, 90% of infected people either overcome the infection or develop latent infection. Other risk factors for LTBI include having immunocompromised conditions such as human immunodeficiency virus (HIV) infection, using immunosuppressant medication, patients on hemodialysis, cancer patients, patients with diabetes, and consuming alcohol and cigarettes [3,4].

LTBI is a common finding among HCWs in the Middle East region. Other risk factors that increase the risk of developing LTBI among HCWs include direct contact with infected patients, location of duties such as in tuberculosis units, microbiology labs, and emergency rooms, low compliance to infection control measures, and being a laboratory personnel who is handling infected materials. To achieve the World Health Organization's "End TB Strategy" which targets to reduce the incidence and tuberculosis-related mortality to 90% and 95%, respectively, by the year 2035, strong screening programs, infection prevention protocols, and a course of antituberculosis drug medication for months are needed [4,5].

Healthcare personnel are at risk of developing LTBI due to their increased frequency of direct patient contact. A systematic study in 2006 by Joshi et al. performed in low- and middle-income groups showed an LTBI prevalence of 54% among HCWs [6]. Another systematic study and review analysis by YektaKooshali et al. in 2019 reported a 27.13% prevalence of LTBI among Iranian HCWs [7].

A systematic review and meta-analysis study by Nasreen et al. in 2016 also showed a 47% total pooled prevalence of LTBI among HCWs [8]. In a systematic review by Apriani et al. in 2019 to measure LTBI prevalence among HCWs in low- and middle-income countries with a total of 32,630 participants, the prevalence ranged between 14% and 98% with a mean prevalence of 49% [9]. In a previous study by Kofi et al. in 2020 at Prince Sultan Military Medical City (PSMMC), to measure LTBI prevalence among newly hired HCWs (two years) with a total of 6,404 subjects who were tested using tuberculin skin test (TST), the total prevalence of LTBI was 4.9% [10].

In Saudi Arabia, health institutions oblige with the Saudi Central Board for Accreditation of Healthcare Institutions. HCWs who are working in critical areas are mandated to undergo annual screening for LTBI. The high prevalence of LTBI in HCWs supported by the above literature enabled our team to work on improving screening practices for the early detection of LTBI in HCWs. Therefore, our study aims to estimate the prevalence of LTBI among nurses working in critical areas at PSMMC, Riyadh, Saudi Arabia.

## Materials and methods

A record-based cross-sectional survey was conducted to measure the prevalence of LTBI among nurses working in high-risk areas at PSMMC, Riyadh, Saudi Arabia, after obtaining approval from the Institutional Review Board of the said institution (approval number: 1645). Occupational health records of all nurses working in high-risk areas from June 1, 2021, to June 1, 2022, were reviewed. The data were recorded throughout the year in the Occupational Health Department of PSMMC. Data gathered included age, gender, nationality, job title, working years, working location, and TST results.

A baseline questionnaire must be filled out before TST is administered. The Mantoux test is the recommended TST with an injection of 0.1 mL containing 5 tuberculin units (TU) of purified protein derivative material intradermally into the volar surface of the forearm using a tuberculin syringe. A follow-up within 48-72 hours post administration is mandatory to interpret the result. If the subjects fail to attend for results reading, the test is considered not valid and must be repeated. A TST ruler is used to measure the induration between the two points in millimeters.

According to the Centers for Disease Control and Prevention recommendations, the interpretation of TST is as follows: a TST reaction of >5 mm is considered positive for patients with recent contact with confirmed infectious tuberculosis cases, immunocompromised patients, individuals using immunosuppressants, and HIV patients. A reaction of ≥10 mm is considered positive for those who are from countries where tuberculosis is common or in countries with high infection rates and people who live or work in high congregate areas such as nursing homes, long-term care facilities, and hospitals. A reaction of ≥15 mm is considered positive for patients without risk factors [11].

The reaction measurement is then recorded in the employee file. Seven hundred and seventy nurses out of 2,025 were included in this study. Inclusion criteria include all the nurses working in critical areas (adult intensive care units, pediatric intensive care units, emergency departments, oncology departments, dialysis departments, tuberculosis labs, isolation rooms, and cardiac center intensive care units), while exclusion criteria include all other hospital departments, other HWCs, and nurses with a previous positive TST.

Data were analyzed using IBM SPSS Statistics for Windows, Version 26.0 (Released 2019; IBM Corp., Armonk, New York, United States). The researchers had access to anonymous data with no personal identifiers such as name, employee number, or medical record number, and the date was numbered with random codes to protect the identity and confidentiality of the staff. The data gathered were protected by a password key, accessible only to the researchers of this paper and only through the safe network of PSMMC. Data collected for this research were not used in other research projects.

## Results

Most of the participants are in the 26-35 age group, with 522 HCWs (67.7%), followed by the 36-45 age group, which includes 151 HCWs (19.6%). The 46-59 age group included 66 HCWs (8.6%), and the lowest group was the 18-25 age group, which accounted for 32 HCWs (4.1%).

Most of the HCWs in this study (511, 66.3%) are from the Philippines, followed by Saudi Arabia and India with 99 HCWs (12.8%) and 88 HCWs (11.4%), respectively. Other nationalities had 73 HCWs, which represents 9.5%. Other nationalities included 55 workers from Malaysia, five workers from Jordan, three workers from Egypt, three workers from the Czech Republic, two workers from Serbia, one worker from Slovakia, one worker from South Africa, one worker from Spain, one worker from Morocco, and one worker from Poland.

In this study, female participants accounted for 679 HCWs (88%) compared to 92 male HCWs (12%). HCWs who have been working at PSMMC for less than five years were 409 HCWs (53%), for 5-10 years were 250 HCWs (32.4%), and for more than 10 years were 112 HCWs (14.6%).

Regarding the working location of HCWs, 126 (16.3%) are working in the adult intensive care unit department, 80 (10.4%) in the pediatric intensive care unit department, 155 (20.1%) in the cardiac intensive care unit department, 144 (18.7%) in the emergency department, 76 (9.9%) in the dialysis department, 140 (18.2%) in the oncology department, and 45 (5.8%) and five (0.6%) in the isolation and tuberculosis lab departments, respectively.

More than half are staff nurse level 2 with 468 nurses (60.7%), followed by staff nurse level 1 with 182 nurses (23.6%), staff nurse level 3 with 86 nurses (11.2%), and charge nurses with 35 nurses (4.5%).

Table 1 represents the sociodemographic data and prevalence of LTBI among study participants. The total prevalence of HCWs with LTBI in our study is 266 HCWs (34.5%). For the prevalence of LTBI among different age groups, the highest prevalence was among older HCWs in the 46-59 age group, with 31 workers (47%) having an LTBI, followed by the 36-45 age group with 65 workers (43%) and the 26-35 and 18-25 age groups with 168 workers (32%) and two workers (6%), respectively; significant associations are found for age groups with a p-value of <0.0002.

**Table 1:** Sociodemographic characteristics of the study population and LTBI prevalence *statistically significant <0.05 (A) Most senior among the nursing staff and report to the charge nurse (B) Report to the staff nurse level 1 and charge nurse (C) Most junior and report to the staff nurse levels 1 and 2 TB: tuberculosis; LTBI: latent tuberculosis infection

Sociodemographic characteristic	Category	Frequency	Rate of positive TB	p-value
Age (in years)	18-25	32 (4.1%)	2 (6%)	0.0002*
	26-35	522 (67.7%)	168 (32%)	
	36-45	151 (19.6%)	65 (43%)	
	46-59	66 (8.6%)	31 (47%)	
Nationality	Saudi	99 (12.8%)	8 (8%)	0.0001*
	Indian	88 (11.4%)	29 (33%)	
	Filipino	511 (66.3%)	203 (40%)	
	Others	73 (9.5%)	26 (36%)	
Gender	Male	92 (12%)	28 (30%)	0.3821
	Female	679 (88%)	238 (35%)	
Working years	Less than five years	409 (53%)	131 (32%)	0.0444*
	Between five and 10 years	250 (32.4%)	85 (34%)	
	More than 10 years	112 (14.6%)	50 (44.6%)	
Department	Adult intensive care unit	126 (16.3%)	34 (27%)	0.0001*
	Pediatric intensive care unit	80 (10.4%)	14 (17.5%)	
	Cardiac intensive care unit	155 (20.1%)	83 (53.5%)	
	Emergency	144 (18.7%)	58 (40.3%)	
	Dialysis	76 (9.9%)	14 (18.4%)	
	Oncology	140 (18.2%)	58 (41.4%)	
	Isolation	45 (5.8%)	4 (8.9%)	
	TB lab	5 (0.6%)	1 (20%)	
Job position	Charge nurse	35 (4.5%)	16 (46%)	0.0001*
	Staff nurse 1^A^	182 (23.6%)	47 (26%)	
	Staff nurse 2^B^	468 (60.7%)	192 (41%)	
	Staff nurse 3^C^	86 (11.2%)	11 (12%)	
Total LTBI prevalence	All nurses in critical areas	266/771 (34.5%)		

Regarding the prevalence of LTBI among each nationality, the highest parentages of LTBI are HCWs from the Philippines with 203 HCWs (40%), followed by other nationalities with 26 HCWs (36%), India with 29 HCWs (33%), and, lastly, Saudi Arabia with eight HCWs (8%); significant associations are found for HCWs' nationality with a p-value of <0.0001. For the prevalence of LTBI in each gender, the female nurses with positive TST results accounted for 238 nurses (35%) of all female nurses, whereas the prevalence in male nurses was found to be 28 nurses (30%) of all male nurses; no significant associations are found for gender with a p-value of <0.3821.

Regarding the working years among nurses and the prevalence of LTBI, nurses who worked for less than five years at PSMMC with positive TST results included 131 workers (32%). Nurses who worked for 5-10 years at PSMMC with positive TST results included 85 workers (34%). Nurses who worked for more than 10 years at PSMMC with positive TST results included 50 workers (44.6%); significant associations are found for working years with a p-value of <0.0444.

Regarding the prevalence of LTBI in relation to the department which HCWs are working, the greatest prevalence of LTBI was among HCWs in the cardiac intensive care unit with more than half of them having LTBI (83 workers, 53.5%). Subsequently, the prevalence in the oncology and emergency departments was 58 workers (41.4%) and 58 workers (40.3%), respectively. The adult intensive care unit prevalence was 34 workers (27%), whereas the pediatric intensive care unit prevalence was 14 workers (17.5%). In the dialysis department, the LTBI prevalence was 14 workers (18.4%), whereas in the tuberculosis lab, the prevalence was one worker (20%). In contrast, the isolation department had the lowest prevalence of LTBI with only four workers (8.9%); significant associations are found for HCWs' working department with a p-value of <0.0001.

Regarding the prevalence of LTBI among different nurses' levels, the highest percentage of LTBI was among charge nurses with 16 positive results (46%), followed by staff nurse level 2 with 192 positive results (41%), then staff nurse level 1 with 47 positive results (26%), and, lastly, staff nurse level 3 with only 11 positive results (12%); significant associations are found for job title with a p-value of <0.0001.

## Discussion

The study aimed to investigate the prevalence of LTBI among nurses working in high-risk hospital departments at PSMMC, Riyadh, Saudi Arabia. Our findings suggest that the study sample was relatively young, with most participants being under 35 years of age (71.7%), and female nurses were predominant in terms of gender (88%), which is consistent with Khamis et al., whose participants were mostly female (67%) and those younger than 40 years old (77%) [2]. Another study by Sabri et al. in Morocco included 631 HCWs, with those who are younger than 45 years old being 76.6% of the total study population and female nurses representing 46% [12]. The prevalence of LTBI in our study is increasing with older age groups. For the 18-25 age group, the prevalence was 6%, and for the 45-59 age group, the prevalence was 47%. These findings showed that LTBI is more common among older HCWs.

Regarding the nurses' nationality, in our study, most nurses came from highly endemic areas, such as the Philippines and India, where higher incidence rates of tuberculosis infection are established. A significant number of Filipino and Indian nurses are working in Saudi Arabia hospitals, which probably has an effect on the overall prevalence in our study. Nurses from the Philippines accounted for 66.3%, and 40% of them had positive TST results. The Saudi nurses (12.8%) had only (8%) with positive TST results. For Indian and other nationality nurses (11.4% and 9.5%, respectively), 33% and 36% of them had positive TST results, respectively. The Saudi nurses have a slightly lower prevalence rate in comparison to the Saudi general population (9.3%) based on Balkhy et al. [13].

In another study done at a tertiary care hospital in Riyadh, Saudi Arabia, by Almohaya et al., a total of 3,024 HCW nurses accounted for 57.7% of all HCWs in this study, and nurses who had positive LTBI using interferon-gamma release assay (IGRA) test were found to be 29.5%; HCWs from the Philippines were found to be 44.3%, with 31.5% having positive LTBI results, followed by HCWs from the Indian subcontinent (23.9%), with an LTBI prevalence of 28.8%, and HCWs from Saudi Arabia (24.8%), with almost 9% having positive LTBI results. However, it is difficult to compare the two studies because of the different testing and inclusion methods on job category and nationality [14].

The LTBI prevalence in our participants was 34.5%. Different studies in the region had different results. Almohaya et al. reported LTBI with a prevalence of 24.2% [14]. In Oman, Khamis et al. reported a prevalence of LTBI among HCWs at 33.2% [2]. These differences were probably because of the heterogeneity of the participants and the screening tools used. As regards the prevalence of LTBI in the different hospital departments in comparison with a study done by Almohaya et al., emergency department workers with positive LTBI in their study were found to be 28.9% compared to 40.3% in our study, whereas in the tuberculosis labs, the prevalence of LTBI was 20% in both studies. In addition, the isolation department was the lowest in the prevalence of LTBI in both studies, with only 8.9% in our results compared to 12.5% in their study [14]. The lowest prevalence was among workers in the isolation department; this most likely was the result of robust compliance with the protective measures for infection control protocols.

To discover how prevalent LTBI was among newly hired HCWs after less than two years of work in four of the major tertiary hospitals in Riyadh, Saudi Arabia, Abbas et al. enrolled 2,650 HCWs in his research, and only 11% were found to be TST positive. This finding can explain that a longer HCW exposure increases the LTBI incidence [15].

## Conclusions

LTBI remains a prevalent issue worldwide and in the Middle East region. HCWs are at the greatest risk of acquiring tuberculosis infection, particularly workers in high-risk departments, older HCWs, and workers from highly endemic countries. Underscoring the need for comprehensive pre-hiring screening, annual screening, affirming infection control protocols, and practicing active management for HCWs with LTBI are required to assist in LTBI eradication.
